# Novel Aspects of the SubA Subunit of the Subtilase Cytotoxin

**DOI:** 10.3390/toxins14020156

**Published:** 2022-02-21

**Authors:** Katharina Sessler, Herbert Schmidt, Holger Barth

**Affiliations:** 1 Institute of Pharmacology and Toxicology, University of Ulm Medical Center, D-89081 Ulm, Germany; katharina.sessler@uni-ulm.de; 2 Department of Food Microbiology and Hygiene, Institute of Food Science and Biotechnology, University of Hohenheim, D-70599 Stuttgart, Germany; herbert.schmidt@uni-hohenheim.de

**Keywords:** AB_5_ toxin, subtilase cytotoxin, SubA, SubB, Shiga toxin, *Escherichia coli*, STEC, retrograde transport, ER, cellular uptake, BiP/GRP78

## Abstract

The subtilase cytotoxin (SubAB) belongs to the family of AB_5_ toxins and is produced together with Shiga toxin (Stx) by certain Stx-producing *E. coli* strains (STEC). For most AB-type toxins, it is assumed that cytotoxic effects can only be induced by a complete holotoxin complex consisting of SubA and SubB. However, it has been shown for SubAB that the enzymatically active subunit SubA, without its transport and binding domain SubB, induces cell death in different eukaryotic cell lines. Interestingly, the molecular structure of SubA resembles that of the SubAB complex. SubA alone is capable of binding to cells and then being taken up autonomously. Once inside the host cell, SubA is transported, similar to the SubAB holotoxin, via a retrograde transport into the endoplasmatic reticulum (ER). In the ER, it exhibits its enzymatic activity by cleaving the chaperone BiP/GRP78 and thereby triggering cell death. Therefore, the existence of toxic single SubA subunits that have not found a B-pentamer for holotoxin assembly might improve the pathogenic potential of subtilase-producing strains. Moreover, from a pharmacological aspect, SubA might be an interesting molecule for the targeted transport of therapeutic molecules into the ER, in order to investigate and specifically modulate processes in the context of ER stress-associated diseases. Since recent studies on bacterial AB_5_ toxins contributed mainly to the understanding of the biology of AB-type holotoxins, this mini-review specifically focus on that recently observed single A-effect of the subtilase cytotoxin and addresses whether a fundamental shift of the traditional AB_5_ paradigm might be required.

## 1. Introduction

Bacterial AB_5_ toxins are a well-studied protein toxin family, which includes medically relevant members, such as Shiga toxin (Stx), cholera toxin (Ctx), pertussis toxin (Ptx), and the most recently discovered, the subtilase cytotoxin (SubAB). The latter is produced by certain Stx-producing *E. coli* (STEC) strains, which are negative for the *eae* gene that is located in the locus of enterocyte effacement (LEE) [1]. STEC strains are mainly found in the gastrointestinal tract of ruminants, but wild animals can also serve as an STEC reservoir or spillover host of STEC, and humans can become infected via the food chain or contaminated water [2]. The particular group of *eae*-negative, *subAB*-positive STEC has been reported only rarely to be associated with human disease and has been mainly isolated from ruminants and foods [3,4,5]. All members of the AB_5_ toxin family consist of a catalytically active A subunit that disrupts essential host cell signaling pathways and a B-pentamer responsible for host cell binding and subsequent uptake of the toxin into the cell [6,7]. Although this general AB_5_ structure and function is conserved in these toxins, the individual members of the AB_5_ toxin family differ in their sequence homology and the enzyme specificities of their A subunits. Until now, it was a major scientific paradigm that only a complete AB_5_ holotoxin complex can cause cytotoxicity. However, recent publications show that the A subunit of the subtilase cytotoxin (SubA) can cause cell death in the absence of its corresponding B subunit (SubB) [8,9].

## 2. Molecular Structure of the Enzyme Subunit SubA

SubAB was discovered during an outbreak of hemolytic-uremic syndrome (HUS) in south Australia in 1998 in the LEE-negative STEC O113:H21 strain 98NK2. Paton et al. originally described a subtilase-like serine protease with AB_5_ structure and termed it subtilase cytotoxin [10]. The corresponding *subAB* genes were positioned close together on the mega-plasmid pO113 and co-transcribed, suggesting an operon structure. The pO113-located *subA* gene comprises 1043 nucleotides and is separated by 16 nucleotides from the 426-bp open reading frame of *subB* [10]. These genes encode a 347-amino-acid-containing A subunit and a 141-amino-acid-containing B subunit. Later, SubAB coding genes were found in numerous other LEE-negative STEC strains [11,12,13,14,15,16]. Paton et al. analyzed the distribution of SubAB in 68 STEC strains isolated from patients with HUS and/or diarrheal disease or from contaminated food linked to an outbreak of HUS, and of 68 STEC strains tested, *subAB* genes were found in 32, including representatives of serogroups O23, O48, O82, O91, O111, O113, O123, O128, O157, OX3, and O non-typeable strains [10].

**Figure 1:**
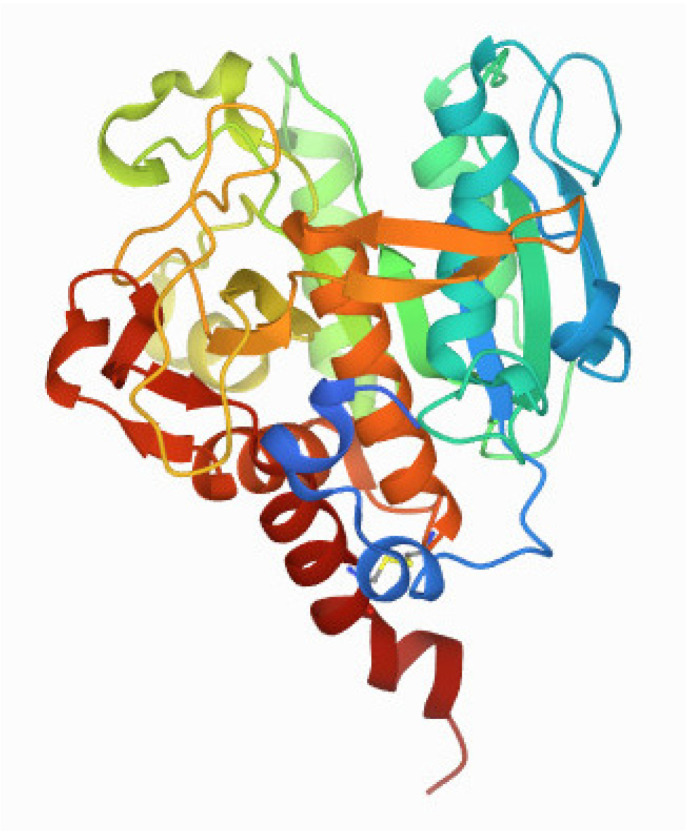
3D view of the enzyme subunit SubA of the subtilase cytotoxin (PDB DOI: 10.2210/pdb4BWG/pdb (Protein Data Bank Nature New Biology 233:223) from primary publication Le Nours et al. [17]. The A2 domain is the dark red helix; the rest of the structure represents the A1 domain. Explanations are given in the text.

Thus far, two major groups of SubAB proteins have been identified: SubAB1, which is encoded on megaplasmids, and SubAB2, which can be further distinguished into the three subtypes of SubAB2-1, SubAB2-2, and SubAB2-3, which are encoded on the bacterial chromosome. SubAB1 and SubAB2 share 90% sequence identity, whereas the three subtypes of SubAB2 are even more conserved, sharing 99% sequence identity [11,12,15,16]. The subtilase cytotoxin established a new family within the AB_5_ toxins due to its distinct subtilase-like serine protease activity via the A subunit [10,17,18]. The *subA* gene encodes a protein (Figure 1) that displays homology to members of the subtilisin S8A superfamily. It has been shown that after its delivery into the endoplasmic reticulum (ER), SubA cleaves the ER-specific chaperone BiP/GRP78 at a single site [19,20]. SubA is composed of two domains, namely the A1 and the A2 domain, which are connected by a disulfide bond by the residues Cys-288 and Cys-331 [17]. The A1 domain harbors the proteolytic activity via a catalytic triad of Asp-52-His-89-Ser-272 [10,17]. The A2 domain, which consists of a helix of about 25 amino acids, penetrates into the B-pentamer mainly via the N-terminal residues Lys-333, Lys-334, Tyr-335, Ile-336, Pro-337, and Val-338, thus forming a SubAB complex [10,17]. Unlike other subtilase family proteases, SubA shows a remarkable substrate specificity due to a unique, deep active site cleft formed by the amino acid residues 81–88 and 234–239 [17]. Moreover, SubA essentially requires serine at position 272 to induce cytotoxicity [17,18]. Interestingly, the overall structure of SubA in the isolated form is very similar to the complex form of SubAB due to a conserved disulfide bond holding the A1 and A2 domains together [17].

## 3. Cellular Uptake and Cytotoxic Activity of the Enzyme Subunit SubA in the Absence of the Transport Subunit SubB

Considering the roles of the subunits for the holotoxin SubAB, the B subunit is responsible for cell binding and uptake. However Western blot experiments by Funk et al. showed a concentration-dependent SubA binding to HeLa cells in the absence of SubB [8]. Cell surface binding of SubA was confirmed by flow cytometry [9], but to the best of our knowledge, a specific SubA receptor has yet not been identified. Since BiP/GRP78, the molecular substrate of SubA protease in the ER, is also present at the cell surface [20], it can be speculated that cell surface bound GRP78 might be a potential SubA receptor. However, further investigations are required to prove this hypothesis. For identification of a putative SubA receptor, a classical pull-down assay could be performed. Alternatively, a genome-wide CRISPR/Cas9 knockout screen for SubA-induced cell death, similar to how it was performed for SubAB, where *O*-glycans were identified as a further SubAB receptor [21], could provide new information. After binding of SubA, it is autonomously taken up into the host cell and causes similar cell morphological changes in several eukaryotic cell lines similar to SubAB [9]. However, cell death induced with SubA alone required higher concentrations of SubA compared to the amount of SubA in the SubAB holotoxin [9]. In vitro and in vivo experiments by Paton et al. identified the endoplasmic Hsp70 chaperone BiP/GRP78 as cellular target for SubAB. Detailed investigations have shown that SubAB-induced cytotoxicity is based on a specific single-site cleavage of BiP/GRP78 in the hinge region connecting its N-terminal ATPase and C-terminal protein-binding domains and that only a single amino-acid substitution in the BiP/GRP78 target was sufficient to protect cells from the toxin effect [19]. In addition, co-localization studies of SubAB and BiP/GRP78 and inhibitor experiments with brefeldin A and phenylarsine lead to the conclusion that SubAB is transported via a clathrin-dependent retrograde pathway to its target compartment the ER [22]. However, the detailed mechanisms underlying the internalization of SubAB might be dependent on the investigated cell line or cell type since the uptake of SubAB by HeLa cells exploits an actin- and lipid raft-dependent pathway but does not require a clathrin-, caveolin-, and dynamin-dependent pathway as shown in an approach with various specific pharmacological inhibitors [23]. By investigating the intracellular retrograde trafficking after the internalization of SubAB in more detail, Smith et al. identified a novel intra-Golgi retrograde trafficking pathway containing the conserved oligomeric Golgi (COG) vesicular tethering factor, the GTPase Rab6, and the ß-COP protein COPI, which is used by SubAB for rapid delivery to the ER [24]. 

**Figure 2:**
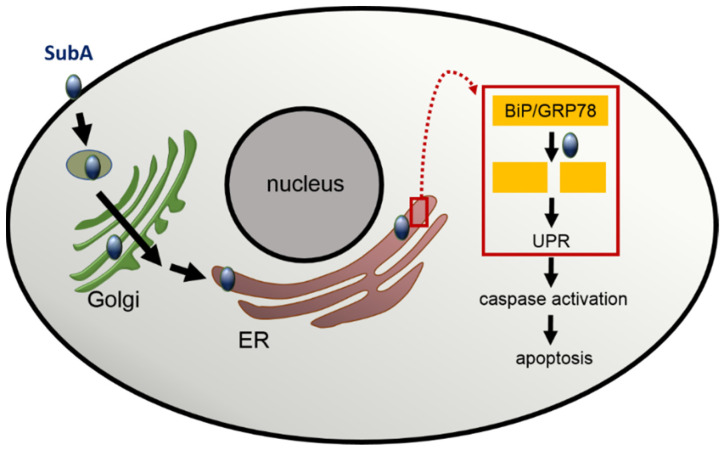
Scheme representing the current model for the uptake, intracellular transport, and cytotoxic effect of SubA in the absence of SubB. Explanations are given in the text.

BiP/GRP78 is a member of the Hsp70 chaperon family and a master regulator of the ER stress-response, as its proper function plays a crucial role in the unfolded protein response (UPR) [25]. In brief, an accumulation of unfolded proteins triggers the disassembly of BiP/GRP78 from the UPR sensor proteins in the ER. This is the prerequisite for a series of events including the activation of the transcription factor 6 (ATF6) and the activation of enzymes like inositol-requiring enzyme 1 (IRE1) and PKR-like ER kinase (PERK), which further activates downstream pathways to re-establish ER homeostasis. However, if the ER stress is too strong and persistent, ER stress signal pathways are activated, which results in apoptosis of the cells [26]. The BiP/GRP78 cleavage after treatment of cells with SubAB triggers ER stress by activating all three ER stress signaling pathways, and due to the persisting SubA activity in the ER, BiP/GRP78 cannot be recovered, leading to a continues ER stress response, which ultimately results in cell death [27,28]. Interestingly, treatment of cells with SubA in the absence of SubB also induced apoptosis [8]. Fluorescence microscopy studies visualized that SubA alone, without SubB being applied to the cells, is able to enter cells and reaches the ER. In the ER, SubA exhibits its enzyme activity and cleaves BiP/GRP78. However, BiP/GRP78 cleavage was delayed when cells were treated with SubA alone compared to SubAB [8,9]. To reach the ER, SubA alone seems to take the same overall route as SubAB via the retrograde trafficking pathway [9] (Figure 2). An experimental hint for this conclusion was the finding that the SubA-catalyzed BiP/GRP78 cleavage was prevented by pre-treatment of cells with brefeldin A, a compound that inhibits Golgi to ER transport [9]. Furthermore, cell death caused by treatment of cells with SubA alone is triggered through an activation of caspase 3 and 7, and the pharmacological inhibition of these caspases reduced SubA-induced cytotoxicity when cells were incubated with SubA in the absence of SubB [8]. Since it was believed that SubB is responsible for guiding the toxin into the ER, the recent results revealed that SubA itself harbors an ER-targeting signal sequence motif, which is the SEEL motif [9]. For cholera toxin, another AB_5_ toxin, a C-terminal KDEL motif in the A subunit, ensures retrograde transport [29]. In the SubA amino acid sequence, a classical KDEL motif is missing; however, the C-terminally located SEEL motif was identified as a crucial ER-targeting signal [9]. However, further detailed investigation is required to elucidate whether SubA alone exploits the same molecular mechanisms for its uptake into cells and its subsequent transport from the Golgi apparatus to the ER as described before for the SubAB holotoxin.

## 4. Potential Exploitation of SubA for the Targeted Delivery of Therapeutic Molecules into the ER

Because of their unique abilities to efficiently deliver enzyme subunits into the cytosol or into specific organelles of mammalian cells without harming the cells during the transport process, various bacterial AB-type toxins have been established as transport systems to deliver “foreign” proteins into cells. This is particularly relevant since many therapeutic proteins, enzymes, and peptides cannot be used for therapy because they are not taken up into cells and therefore do not reach their intracellular drug targets. Thus, amongst others, non-toxic mutants and portions of anthrax toxin, diphtheria toxin, and *C. botulinum* C2 toxin became attractive tools for delivering pharmacologically active cargo (macro)molecules into the cytosol of their target cells. [30,31,32,33,34]. Recently, poly(D,L-lactide-co-glycolic) acid (PLGA) nanoparticles were fused to SubAB to target macrophages and to examine anti-inflammatory effects [35]. Since SubA, in contrast to the before mentioned toxins, does not translocate into the cytosol but stays in the ER, it should represent a specific transporter to deliver therapeutic cargo molecules into the ER, e.g., for the targeted modulation of ER stress-induced processes in the context of ER stress-associated diseases, such as metabolic and neuronal diseases or cancer [36]. For generation of such transporter molecules, a non-toxic SubA protein, where the essential serine residue at position 272 is substituted, would be required, and the cargo molecules would be coupled by genetic fusion [34] or biotin/avidin technology. In the latter approach, a recombinant SubA-avidin fusion protein serves for cellular delivery via the SubA portion and for binding of biotinylated cargo molecules via the avidin platform as established for other toxin-based transporter molecules [33].

## 5. Discussion

In summary, SubA alone demonstrates a similar cellular uptake and cytotoxic mode of action on eukaryotic cells as the SubAB holotoxin. However, SubA uptake and/or its transport into the ER seems to be less efficient in the absence of SubB. This suggests that SubB is not essential to cause cytotoxicity, but the presence of SubB amplifies the cytotoxic effect tremendously, which might enable the toxin to act at lower concentrations. Thus, the existence of toxic, single SubA subunits, which were released from the bacteria but have not found a SubB-pentamer for holotoxin assembly, might broaden and enhance the pathogenic potential of subtilase-producing *E. coli* strains.

In vivo studies showed that intraperitoneal injection of SubAB caused microangiopathic hemolytic anemia, thrombocytopenia, and renal impairment in mice [37]. This pathology is similar to animal studies with purified Stx [38,39,40] and illustrates parallels to human cases of STEC-associated HUS [41,42]. Furthermore, a recent study demonstrated that the co-injection of Stx2 and SubAB in mice resulted in a more severe disease progression compared to mice treated with only one toxin, highlighting the contribution of SubAB in STEC infection [43]. Thus far, all studies regarding the single-A effect of the subtilase cytotoxin have been performed with cultured cells. Therefore, it would be interesting to investigate if not only SubAB but also SubA could induce HUS-like symptoms in mice. In that context, studies on the effect SubA in human intestinal organoid models, so-called “miniguts,” would be of particular interest. Besides the subtilase toxin, STEC strains produce also Stx, which is considered to be the more clinically relevant toxin. Stx belongs also to the AB_5_ toxin family and is structured similarly to the subtilase toxin, which guides us to the hypothesis that the A subunit of Stx, without its corresponding B subunit, might also induce cytotoxicity. A recent publication addressed this question and cytotoxicity assays revealed that the StxA2a subunit induced comparable cytotoxic effects similar to the recombinant Stx2a-holotoxin on HeLa cells and even higher cytotoxic effects on Vero cells [44]. However, detailed mechanisms of uptake and intracellular transport are still unknown and need to be investigated. Since the A subunits of SubAB and Stx2 induce cytotoxicity independent of their corresponding B subunits, the single-A effect might play a yet unknown role in STEC-associated diseases.

## Data Availability

Not applicable.
